# Multicenter study on clinical outcomes and poor prognostic factors in patients with *Klebsiella pneumoniae* bacteremia receiving cefoperazone/sulbactam treatment

**DOI:** 10.1007/s10096-024-04892-x

**Published:** 2024-07-12

**Authors:** Tsung-Ta Chiang, Ming-Hsien Chiang, Hung-Jen Tang, Zhi-Yuan Shi, Mao-Wang Ho, Chia-Hui Chou, Shang-Yi Lin, Po-Liang Lu, Ting-Shu Wu, Shian-Sen Shie, Jien-Wei Liu, Feng-Yee Chang, Yin-Ching Chuang, Fu-Der Wang, Ya-Sung Yang

**Affiliations:** 1 Division of Infectious Diseases and Tropical Medicine, Department of Internal Medicine, Tri- Service General Hospital, National Defense Medical Center, No. 325, Section 2, Cheng-Kung Road, Taipei, 11490 Taiwan; 2 Department of Nutritional Sciences, Fu-Jen Catholic University, New Taipei City, Taiwan; 3 Department of Medicine, Chi Mei Medical Center, Tainan, Taiwan; 4 Department of Internal Medicine, Taichung Veterans General Hospital, Taichung, Taiwan; 5 Division of Infectious Diseases, Department of Internal Medicine, China Medical University Hospital, Taichung, Taiwan; 6 Division of Infectious Diseases, Department of Internal Medicine, Kaohsiung Medical University Hospital, Kaohsiung, Taiwan; 7 Division of Infectious Diseases, Department of Internal Medicine, Chang Gung Memorial Hospital, Taoyuan, Taiwan; 8 Division of Infectious Diseases, Department of Internal Medicine, Kaohsiung Chang Gung Memorial Hospital, Kaohsiung, Taiwan; 9 Division of Infectious Diseases, Department of Internal Medicine, Taipei Medical University Hospital, Taipei, Taiwan; 10 National Yang-Ming Chiao-Tung University, Taipei, Taiwan; 11 Center for Liquid Biopsy and Cohort Research, Kaohsiung Medical University, Kaohsiung, Taiwan; 12 School of Post-Baccalaureate Medicine, College of Medicine, Kaohsiung Medical University, Kaohsiung, Taiwan; 13 Institute of Biology and Anatomy, National Defense Medical Center, Tipei, Taiwan

**Keywords:** Bacteremia, Breakpoint, Cefoperazone, *Klebsiella pneumoniae*, Minimal inhibitory concentration, Outcome, Sulbactam

## Abstract

**Background:**

Infections caused by *Klebsiella pneumoniae* are common and result in high mortality rates. In vitro studies demonstrated the potency of cefoperazone/sulbactam (CPZ/SUL) against *Klebsiella pneumoniae*. However, the clinical efficacy of CPZ/SUL for the treatment of *K. pneumoniae* bacteremia has not been studied.

**Objectives:**

This study aimed to associate the clinical outcomes of patients with bacteremia with the minimal inhibitory concentrations (MICs) of CPZ/SUL against the causative *K. pneumoniae* isolates.

**Methods:**

This multicenter, retrospective study was conducted in Taiwan between July 2017 and April 2021. Patients with *K. pneumoniae* bacteremia treated with CPZ/SUL were enrolled in this study. CPZ/SUL MICs were determined using the agar dilution method. Data on the patients’ clinical outcomes and characteristics were collected and analyzed.

**Results:**

In total, 201 patients were enrolled. Among the causative *K. pneumoniae* isolates, 180 (89.5%) were susceptible to CPZ/SUL. Most patients (*n* = 156, 77.6%) had favorable outcomes. The 30-day mortality rate was 11.9% (*n* = 24). Multivariate risk analyses showed that higher APACHE II score (Odds Ratio [OR], 1.14; Confidence Interval [CI], 1.07–1.21; *p* < 0.001), metastatic tumors (OR, 5.76; CI, 2.31–14.40; *p* < 0.001), and causative *K. pneumoniae* CPZ/SUL MICs > 16 µg/ml (OR, 4.30; CI, 1.50–12.27; *p* = 0.006) were independently associated with unfavorable outcomes.

**Conclusion:**

Patients with *K. pneumoniae* bacteremia treated with CPZ/SUL at a ratio 1:1 had favorable outcomes when the CPZ/SUL MICs were ≤ 16 µg/ml. Patients with higher APACHE II scores and metastatic tumors had unfavorable outcomes.

**Supplementary Information:**

The online version contains supplementary material available at 10.1007/s10096-024-04892-x.

## Introduction

*Klebsiella pneumoniae* is a common and life-threatening community-acquired, healthcare-associated, and hospital-acquired pathogen. *K. pneumoniae* can cause pneumonia, urinary tract infections, intra-abdominal infections, liver abscesses, bacteremia, and other invasive infections [1–3]. The mortality rates of patients with *K. pneumoniae* bacteremia differed from 20 to 50%, depending on the infected population, and the rates became higher when the severity of the infection increased and the presence of carbapenem resistances [4–6]. The emergence of extended-spectrum β-lactamase (ESBL)-producing *K. pneumoniae* during the past decades has hindered the treatment of these infections and further limited available drug choices for antimicrobial therapy [7–9]. Adequate treatment of patients infected with this problematic pathogen is a major concern to physicians.

Cefoperazone (CPZ) is a third-generation cephalosporin that is active against the most commonly encountered gram-negative bacteria (GNB) [10–12], but not ESBL-producing GNB. The inclusion of sulbactam (SUL), a penicillanic acid sulfone with activity against Ambler class A enzymes, broadened the antimicrobial spectrum of CPZ [13, 14].

Over the past, the emergence of ESBL-producing *K. pneumoniae* has caused a serious clinical burden [7–9]. The Taiwan Surveillance of Antimicrobial Resistance program conducted from 2002 to 2012 reported that the prevalence of ESBL-producing *K. pneumoniae* increased from 4.8 to 11.9% in Taiwan [15]. According to the SENTRY antimicrobial surveillance program data, the prevalence of multidrug-resistant (MDR) *Enterobacterales-*associated bloodstream infections increased from 6.2 to 15.8% between 1997 and 2016 [9].

The CPZ/SUL combination is active against many MDR GNBs, including ESBL-producing *Enterobacterales*, *Pseudomonas aeruginosa*, and *Acinetobacter baumannii* [16, 17]. CPZ/SUL is effective against MDR GNBs that cause febrile neutropenia, intra-abdominal infections, community-acquired pneumonia, and hospital-acquired pneumonia [12, 18–22]. However, there are no available minimal inhibitory concentration (MIC) interpretation breakpoints for the CPZ/SUL combination according to the Clinical and Laboratory Standards Institute (CLSI) and European Committee on Antimicrobial Susceptibility Testing (EUCAST) guidelines [23, 24]. In Taiwan, antimicrobial susceptibility test reports for CPZ/SUL are generated by automated testing using a 2:1 ratio of CPZ to SUL, and the results are interpreted using the CLSI breakpoints for cefoperazone against *Enterobacterales*. Studies have reported that CPZ/SUL administered at a 1:1 ratio has superior antibacterial activities against ESBL-producing *K. pneumoniae* and most MDR GNB compared with the 2:1 ratio [25, 26]. A recent study revealed an 82.7% clinical success rate in treating bacteremia caused by ESBL-producing *Enterobacterales* with 1:1 CPZ/SUL [27].

We conducted a multicenter, retrospective study to correlate the MIC values of a 1:1 ratio of CPZ/SUL against *K. pneumoniae* and the clinical outcomes of patients with *K. pneumoniae* bacteremia.

## Materials and methods

### Study design and patients

This multicenter study was conducted between July 2017 and September 2022 at eight medical centers located in different parts of Taiwan, including Southern Taiwan (Chi Mei Medical Center [CMMC], Kaohsiung Chang-Guan Memorial Hospital [KCGMH], and Kaohsiung Medical University Hospital [KMUH]), Central Taiwan (China Medical University Hospital [CMUH] and Taichung Veterans General Hospital [TCVGH]), and Northern Taiwan (Linkou Chang Gung Memorial Hospital [LCGMH], Tri-Service General Hospital [TSGH], and Taipei Veterans General Hospital [TVGH]).

The enrolled patients were > 20 years of age had monomicrobial bacteremia caused by *K. pneumoniae* and were initially treated using antimicrobial monotherapy with a 1:1 ratio of CPZ/SUL within 24 h of bacteremia onset with treatment lasting for more than 72 h. The onset of bacteremia was defined as the date of index blood culture collected [25, 28]. CPZ/SUL was given intravenously every 12 h at a standard dosage of 2/2 g, with dosage modification as the manufacturer’s guidelines, according to the estimated creatinine clearance using the Cockroft–Gault equation [29]. Patients receiving additional antimicrobial therapies exceeding 48 h were excluded, except those treatments were targeting GPCs, virus or fungi. This study was approved by the Institutional Review Board (IRB) of the TSGH (No. 1-106-05-116) and the IRBs of all other participating hospitals.

### Antimicrobial susceptibility test

The antimicrobial MICs (µg/ml) were determined using the agar dilution method in accordance with CLSI recommendations [23]. The 1:1 combination ratio was used [25]. CPZ/SUL powder was purchased from TTY Biopharm (Taipei, Taiwan).

### Variable definition and assessment of the treatment efficacy

The Charlson Comorbidity Index [30] was used to assess comorbidities. Immunosuppressant therapy indicated those patient receiving prednisolone for at least10mg per day (or equivalent potency agents) from 2 days before bacteremia onset till 30 days post the event. The Acute Physiology and Chronic Health Evaluation II (APACHE II) [31] was used to assess disease severity. The source of bacteremia was classified as respiratory tract infection/pneumonia, urinary tract infection, soft-tissue infections, intraabdominal infection, or primary bloodstream infection, according to the definitions of the Centers for Disease Control and Prevention [32]. Clinical outcomes were assessed at 30 days. The clinical outcomes were recorded and divided into four categories: cure, improvement, lack of efficacy, and death. A cure was defined as the absence of symptoms and signs of infection without the requirement for additional antibiotic therapy and a negative result in the subsequent blood culture within a week of the onset of bacteremia. Improvement indicated that the symptoms and signs subsided with or without laboratory improvement, based on clinical judgment, and further antibiotic treatment was required. Lack of efficacy was defined as clinical progression or persistent bacteremia at the end of CPZ/SUL treatment [33]. Regarding the correlation between treatment efficacy and the MICs of CPZ/SUL, cure and improvement were defined as favorable outcomes. In contrast, a lack of efficacy and death were defined as unfavorable outcomes.

### Statistical analyses

Descriptive statistics were used to present the demographic characteristics of enrolled patients. The demographic characteristics of the two groups (MIC ≤ 16 and MIC > 16) were compared using the Fisher’s exact test for categorical variables. Continuous variables are presented as the mean ± standard deviation and compared using Student’s t-test. Statistical significance was set at *p* < 0.05. A multivariate analysis was performed for all variables that were statistically significant in the univariate analysis. Odds ratios (ORs) with 95% confidence intervals (CIs) and *p*-values were calculated. All statistical analyses were performed using SPSS version 20 (IBM Corp., Armonk, NY, USA).

## Results

**Fig. 1:**
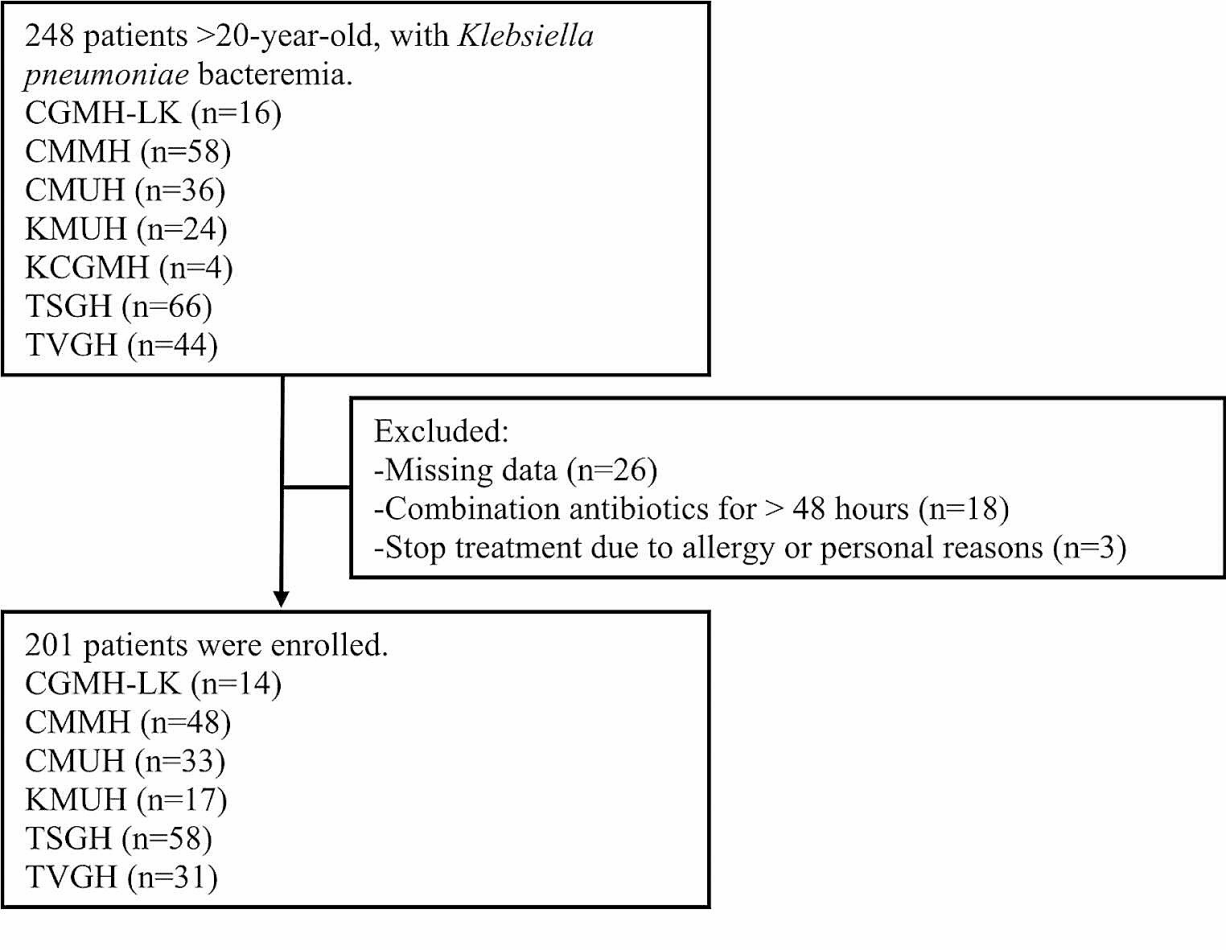
Methodology for application of exclusion criteria. CPZ/SUL, cefoperazone/sulbactam; CGMH-LK, Chang Gung Memorial Hospital; CMMH, Chi Mei Medical Hospital; CMUH, China Medical University Hospital; KCGMH, Kaohsiung Chang Gung Memorial Hospital; KMUH, Kaohsiung Medical University Hospital; TSGH Tri-Service General Hospital; TVGH Taipei Veterans General Hospital; TCTVGH, Taichung Veterans General Hospital

During the study period, 201 patients with *K. pneumoniae* bacteremia were enrolled based on the patient selection criteria (Fig. 1). The patient demographic data were presented in Table 1. The majority of patients were male. The most prevalent comorbidities included diabetes mellitus, impaired liver function, and impaired renal function. The primary cause of bacteremia was intra-abdominal infection, followed by primary bacteremia, respiratory tract infection, and urinary tract infections. Antimicrobial susceptibility results for *K. pneumoniae* are detailed in Table 2.

**Table 1:** Demographic and clinical characteristics of patients with *Klebsiella pneumoniae* bacteremia receiving cefoperazone/sulbactam treatment.^*^

		All	Agar dilution (1:1) MIC		*p* value
			MIC ≤ 16	MIC > 16	
Number		201	180	21	
Sex	Male	124 (61.69)	111 (61.67)	13 (61.90)	0.99
	Female	77 (38.31)	69 (38.33)	8 (38.10)	
Age (Mean ± SD)		68.78 ± 14.86	68.39 ± 15.49	72.14 ± 6.57	0.06
APACHE II score (Mean ± SD)		13.98 ± 6.94	13.42 ± 6.88	18.76 ± 5.80	0.001
Charlson Comorbidity score > 3		156 (77.61)	137 (76.11)	19 (90.48)	0.17
Comorbidities					
Liver function impairment		61 (30.35)	56 (31.11)	5 (23.81)	0.62
Renal function impairment		41 (20.40)	31 (17.22)	10 (47.62)	0.003
Heart failure		15 (7.46)	15 (8.33)	0 (0.00)	0.38
Diabetes mellitus		77 (38.31)	65 (36.11)	12 (57.14)	0.10
Neutropenia		8 (3.98)	8 (4.44)	0 (0.00)	0.99
Immunosuppressant therapy^**^		14 (6.97)	10 (5.56)	4 (19.05)	0.04
Metastatic tumor		35 (17.41)	33 (18.33)	2 (9.52)	0.54
Infection sources					0.48
Respiratory tract		33 (16.42)	27 (15.00)	6 (28.57)	
Urinary tract		28 (13.93)	25 (13.89)	3 (14.29)	
Intra-abdomen		65 (32.34)	60 (33.33)	5 (23.81)	
Primary bacteremia		64 (31.84)	57 (31.67)	7 (33.33)	
Others^***^		11 (5.47)	11 (6.11)	0 (0.00)	
Outcomes					0.001
Favorable		156 (77.61)	146 (81.11)	10 (47.62)	
Cure		62 (30.84)	59 (32.78)	3 (14.29)	
Improvement		94 (46.77)	87 (48.33)	7 (33.33)	
Unfavorable		45 (22.39)	34 (18.89)	11 (52.38)	
Lack of efficacy^****^		21 (10.45)	16 (8.89)	5 (23.81)	
Death		24 (11.94)	18 (10.00)	6 (28.57)	

MIC, mimimal inhibitory concentration

* Data are n (%) unless otherwise stated

** Immunosuppressant therapy: patients receiving prednisolone for at least10mg per day (or equivalent potency agents) from 2 days before bacteremia onset till 30 days post the event

*** Others, other infection sources, six cases of catheter-related blood stream infection (CRBSI) and five cases of soft tissue or wound infection

**** Lack of efficacy was defined as clinical progression or persistent bacteremia at the end of CPZ/SUL treatment

**Table 2:** The antimicrobial susceptibilities of 201 *Klebsiella pneumoniae* isolates

Antimicrobial agents	Susceptibility (*n*/*N*) %		
	S	I	*R*
Amikacin	95.02	1.49	3.48
Gentamicin	76.12	5.97	17.91
Ampicillin	0.00	0.00	100.00
Piperacillin/Tazobactam	74.63	7.46	17.91
Cefazolin	34.33	27.36	38.31
Ceftriaxone	68.66	0.99	30.35
Ceftazidime	65.67	4.48	29.85
Cefepime	83.08	1.00	15.92
Ciprofloxacin	67.66	6.47	25.87
Levofloxacin	73.13	3.48	23.38
Imipenem	90.05	1.99	7.96
Ertapenem	90.05	2.99	6.96
Tigecycline	89.55	5.97	4.48
Trimethoprim/sulfamethoxazole	58.71	0.00	41.29

**Fig. 2:**
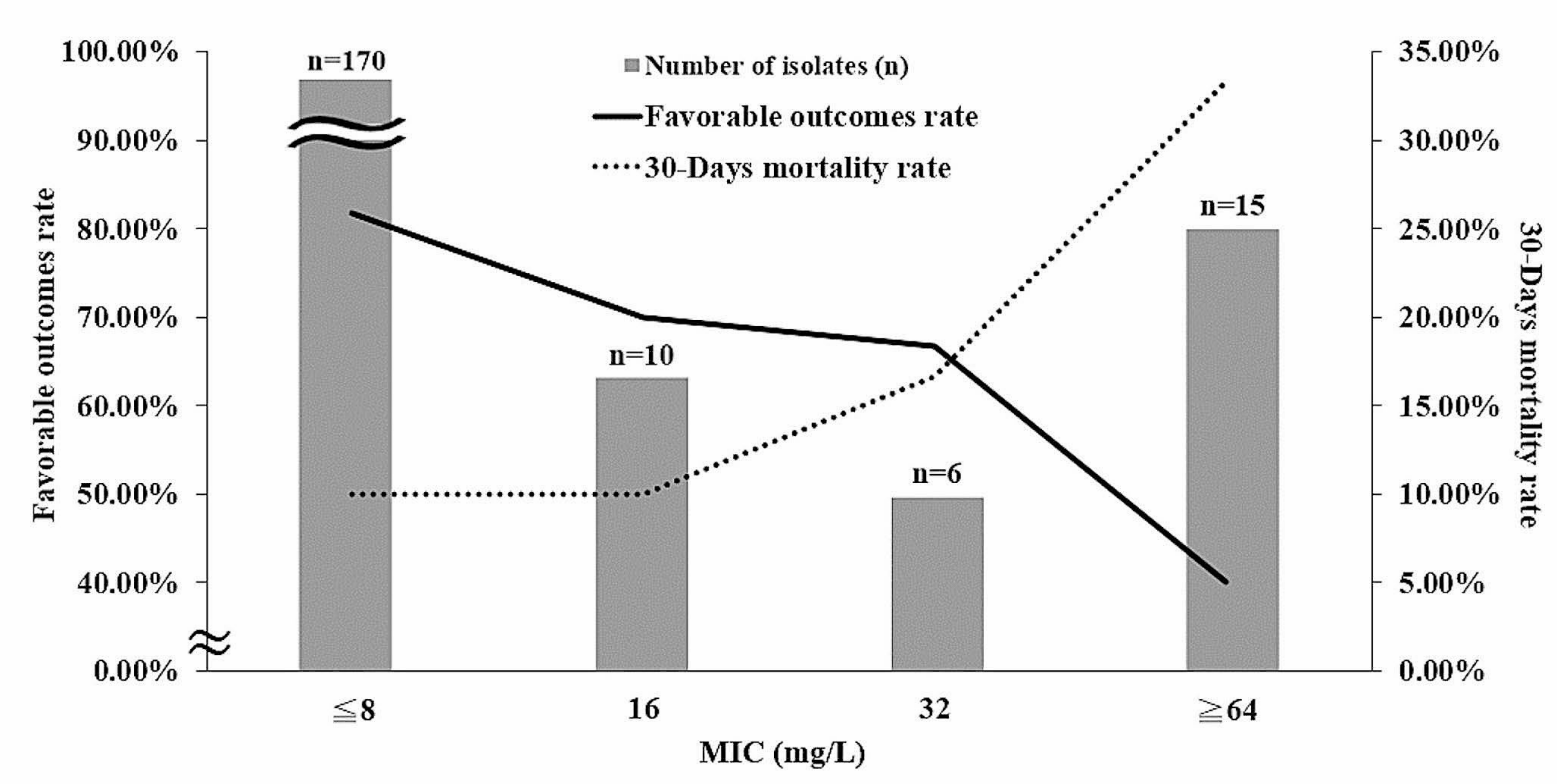
Distribution of the cefoperazone/sulbactam minimum inhibitory concentration (MIC) values and clinical outcomes correlated with cefoperazone/sulbactam minimum MIC values among the 201 *Klebsiella pneumoniae* isolates

The MIC of CPZ alone and the 1:1 combination of CPZ/SUL against *K. pneumoniae* are shown in Table 3. The MIC ranges and MIC_50_ values for CPZ and CPZ/SUL were similar. However, the MIC_90_ values were lower for CPZ/SUL (MIC_90_: 32 ug/ml) than for CPZ (MIC_90_: >64 ug/ml) alone. Among the 201 isolates, 180 (89.55%) were susceptible, six (2.99%) were intermediate, and 15 (7.46%) were resistant to CPZ/SUL. For isolates that were not susceptible to CPZ, the addition of SUL restored the susceptibility rate from 0 to 53.33% and reduced the resistance rate from 82.22 to 33.33% (Table 3). Distribution of the cefoperazone/sulbactam MIC values among those *K. pneumoniae* isolates were showed in Fig. 2. Most of those *K. pneumoniae* isolates in this study exhibited MIC values of less than 8 µg/ml (< 8 µg/ml, *n* = 157, 78.11%) (Fig. 2).

**Table 3:** The minimal inhibitory concentrations and the susceptibilities of cefoperazone alone and in combination with sulbactam (1:1) against *Klebsiella pneumoniae* isolates

*K. pneumoniae* (*n* = 201)	MIC (ug/ml)				Susceptibility [%(*n*)]^a^		
	MIC_50_	MIC_90_	MIC range		S	I	*R*
CPZ	0.25	> 64	0.0625 ~ > 64		77.61% (156)	3.98% (8)	18.41% (37)
CPZ/SUL	0.25	32	0.0625 ~ > 64		89.55% (180)	2.99% (6)	7.46% (15)
CPZnS K. pneumoniae (*n* = 45)	MIC (ug/ml)				Susceptibility [%(*n*)]^a^		
	MIC_50_	MIC_90_	MIC range		S	I	*R*
CPZ	> 64	> 64	32 ~ > 64		0.00% (0)	17.78% (8)	82.22% (37)
CPZ/SUL	16	> 64	2 ~ > 64		53.33% (24)	13.33% (6)	33.33% (15)

^a^The susceptibility breakpoints were adapted from Clinical and Laboratory Standards Institute 2019 for cefoperzaone against *Enterobacterales*: S, MIC ≤ 16 mg/L; I, MIC = 32 mg/L; R, MIC ≥ 64 mg

CPZnS, cefoperazone-non-susceptible; CPZ, cefoperazone; SUL, sulbactam; CPZ/SUL, cefoperazone/sulbactam; MIC, minimal inhibitory concentration

Outcome evaluations revealed that 77.61% exhibited favorable outcomes (cure and improvement) and 22.39% showed unfavorable outcomes (death and lack of treatment efficacy). (Table 1). The clinical outcomes correlated with CPZ/SUL MIC values were showed in Fig. 2. As the MIC value increased, the rate of favorable outcomes decreased, and the 30-days mortality rate increased.

Comparing the patient characteristics and outcomes in causative *K. pneumoniae* isolates with CPZ/SUL MIC ≤ 16 µg/ml and > 16 µg/ml in Table 1, we observed that unfavorable outcomes were more frequent in those with MIC > 16 µg/ml than those with MIC ≤ 16 µg/ml. Those infected by isolates with MIC > 16 µg/ml had a higher APACHE II scores and higher prevalence of impaired renal function. There were no significant differences in sex, age, or source of infection between the two groups.

Logistic regression analysis of the prognostic factors for unfavorable outcomes was shown in Table 4. Comparing the two groups, patients with higher APACHE II score (mean ± standard deviation, 12.74 ± 6.35 vs. 18.27 ± 7.18 points; *p* < 0.001), metastatic tumors (*n* = 19, 12.18% vs. *n* = 16, 35.56%; *p* < 0.001), and infection by *K. pneumoniae* isolates with CPZ/SUL MIC > 16 µg/ml (*n* = 10, 6.41% vs. *n* = 11, 24.44%; *p* = 0.001) were associated with a higher risk of unfavorable outcomes in univariate analysis. In multivariate analysis, patients with a higher APACHE II score (OR, 1.14; 95% CI, 1.07–1.21; *p* < 0.001), metastatic tumors (OR, 5.76; CI, 2.31–14.40; *p* < 0.001), and infection by *K. pneumoniae* isolates with CPZ/SUL MIC > 16 µg/ml (OR, 4.30; CI, 1.50–12.27; *p* = 0.006) were independently associated with unfavorable outcomes.

**Table 4:** Univariate and multivariate logistic analyses of factors associated with unfavorable outcomes in patients with *Klebsiella pneumoniae* bacteremia.^*^

		Univariant analysis				Multivariant analysis	
		Favorable outcomes	unfavorable outcomes	OR (95%CI)	*p* value	OR (95%CI)	*p* value
Number		156	45				
Sex	Male (n, %)	96 (61.54)	28 (62.22)	1.03 (0.52–2.04)	0.93	0.81 (0.35–1.90)	0.63
	Female (n, %)	60 (38.46)	17 (37.78)				
Age (Mean ± SD)		68.52 ± 15.48	69.69 ± 15.00	1.01 (0.98–1.03)	0.64	0.99 (0.95–1.02)	0.45
APACHE II score (Mean ± SD)		12.74 ± 6.35	18.27 ± 7.18	1.12 (1.07–1.18)	**< 0.001**	1.14 (1.07–1.21)	**0.002**
Charlson Co-morbidities score > 3 (n, %)		117 (75.00)	39 (86.67)	2.17 (0.85–5.51)	0.10	1.02 (0.83–1.02)	0.83
Co-morbidities (n, %)							
Liver function impairment		45 (28.85)	16 (35.56)	1.36 (0.68–2.75)	0.39		
Renal function impairment		33 (21.15)	8 (17.78)	0.81 (0.34–1.90)	0.62		
Heart failure		12 (7.69)	3 (6.67)	0.86 (0.23–3.18)	0.82		
Diabetes mellitus		55 (35.26)	22 (48.89)	1.76 (0.90–3.43)	0.10		
Neutropenia		7 (4.49)	1 (2.22)	0.49 (0.06–4.04)	0.50		
Immunosuppressant therapy^**^		12 (7.69)	2 (4.44)	0.56 (0.12–2.59)	0.46		
Metastatic tumor		19 (12.18)	16 (35.56)	3.98 (1.83–8.65)	**< 0.001**	5.15 (1.10-24.06)	**0.03**
Infection sources (n, %)							
Respiratory tract		18 (11.54)	15 (33.33)	2.98 (1.20–7.36)	0.02	1.67 (0.58–4.81)	0.34
Urinary tract		23 (14.74)	5 (11.11)	0.78 (0.25–2.41)	0.78	0.61 (0.17–2.18)	0.45
Intra-abdomen		58 (37.18)	7 (15.56)	0.43 (0.16–1.15)	0.09	0.40 (0.13–1.23)	0.11
Primary bacteremia (Ref.)		50 (32.05)	14 (31.11)	1	-	1	-
Others^***^		7 (4.49)	4 (8.89)	2.04 (0.52–7.98)	0.31	2.63 (0.54–12.83)	0.23
MIC > 16 ug/ml		10 (6.41)	11 (24.44)	4.72 (1.86–12.02)	**0.001**	4.37 (1.49–12.83)	**0.007**

MIC, minimal inhibitory concentration

* Data are n (%) unless otherwise stated

** Immunosuppressant therapy: patients receiving prednisolone for at least10mg per day (or equivalent potency agents) from 2 days before bacteremia onset till 30 days post the event

*** Others, other sources, included six cases of catheter-related blood stream infection (CRBSI) and five cases of soft tissue or wound infection

## Discussion

This is the first multicenter study to investigate the effects of CPZ/SUL therapy in patients with *K. pneumoniae* bacteremia and to provide reference clinical breakpoints and prognostic factors for outcomes. The correlation analysis of the MIC values of CPZ/SUL against *K. pneumoniae* with the clinical outcomes revealed that most patients (81.1%) infected by isolates with MIC ≤ 16 µg/ml had favorable outcomes. MIC values > 16 µg/ml were independently associated with unfavorable outcomes. In addition, higher APACHE II scores and metastatic tumors were associated with unfavorable outcomes in patients with *K. pneumoniae* bacteremia.

The major mechanism underlying third-generation cephalosporin resistance in *K. pneumoniae* in Taiwan and worldwide is the presence of ESBL genes [34, 35]. The consumption of carbapenems has increased, which has promoted the spread of carbapenem-resistant *K. pneumoniae* [36, 37]. Therefore, CPZ/SUL may offer a valuable carbapenem-sparing alternative for effectively covering ESBL producers, which is important in antimicrobial stewardship [27]. However, in areas where the prevalence of carbapenem resistance is significant, the empirical use of CPZ/SUL should be approached with caution [38, 39].

The addition of SUL effectively restored the efficacy of CPZ from 77.6 to 89.6%. Even in isolates that were not susceptible to CPZ, the addition of SUL to CPZ restored antimicrobial susceptibility from 0 to 53% in the current study (Table 3). In most previous studies, the ratio of CPZ to SUL was 2:1 [40–43]. Recent studies have established that CPZ/SUL ratio of 2:1 and 1:1 significantly increased the efficacy against ESBL strains and MDR GNB compared with CPZ alone [25]. Moreover, CPZ/SUL at a 1:1 ratio produced better activity against MDR GNB than that at a 2:1 ratio [26].

To date, no CLSI clinical breakpoints have been reported for CPZ/SUL in *K. pneumoniae*. The CLSI CPZ breakpoints for *Enterobacterales* (MIC ≤ 16 µg/ml, susceptible; MIC = 32 µg/ml, intermediate; MIC ≥ 64 µg/ml, resistant) [23] are often used to interpret susceptibility results for CPZ/SUL. Despite the emergence of resistance, CPZ/SUL maintains good antimicrobial efficacy. A large-scale study in China from 2010 to 2018 reported susceptibility rates of *K. pneumoniae* to CPZ/SUL (2:1) ranging from 72.1 to 76.9% [44]. In the current study, the susceptibility was even higher (89.6%) when using the 1:1 CPZ: SUL combination. CPZ/SUL MIC > 16 µg/ml was associated with unfavorable outcomes, indicating that patients with bacteremia who were infected with *K. pneumoniae* isolates with MIC > 16 µg/ml should not be treated with CPZ/SUL. In contrast, most patients with CPZ/SUL MIC ≤ 16 µg/ml exhibited favorable outcomes (81.1%), indicating the efficacy of treatment with 1:1 CPZ/SUL.

In the current study, we observed that the APACHE score and the presence of metastatic tumors were significant risk factors for unfavorable outcomes. These findings were consistent with prior investigations on *K. pneumoniae* bacteremia [45, 46]. Therefore, judicious antimicrobial treatment is crucial for patients with such risk factors. When the MICs ≤ 16 µg/ml, CPZ/SUL could be confidently used to treat *K. pneumoniae* bacteremia. It is also important to follow the results of antimicrobial susceptibilities and following antimicrobial stewardship principles.

This study had some limitations. The major limitations are its retrospective design with potential intrinsic selection bias, and the fact that the detailed treatment course cannot be controlled. Further randomized controlled studies are required to confirm these findings. The strengths of this study include the inclusion of a relatively large number of patients from multiple medical centers located in representative regions of Taiwan using stringent inclusion criteria. Our findings provide clinicians with useful information regarding the outcomes and risk factors of patients with *K. pneumoniae* bacteremia treated with CPZ/SUL.

## Conclusion

Patients with *K. pneumoniae* bacteremia treated with CPZ/SUL at a ratio of 1:1 had a favorable outcome when the CPZ/SUL MICs were ≤ 16 µg/ml. Higher APACHE II scores and metastatic tumors were associated with unfavorable outcomes.

### Electronic supplementary material

Below is the link to the electronic supplementary material.

Supplementary Material 1

## Data Availability

No datasets were generated or analysed during the current study.

## References

[1] Liao CH, Huang YT, Hsueh PR (2022) Multicenter surveillance of capsular serotypes, virulence genes, and antimicrobial susceptibilities of *Klebsiella pneumoniae* causing bacteremia in Taiwan, 2017–2019. Front Microbiol 13:783523 35369508 10.3389/fmicb.2022.783523 PMC8971976

[2] Magill SS, O’Leary E, Janelle SJ et al (2018) Changes in prevalence of health care-associated infections in U.S. hospitals. N Engl J Med 379:1732–1744 30380384 10.1056/NEJMoa1801550 PMC7978499

[3] Restuccia PA, Cunha BA, Klebsiella (1984) Infect Control 5:343–347 6564087 10.1017/S0195941700060549

[4] Tumbarello M, Viale P, Viscoli C et al (2012) Predictors of mortality in bloodstream infections caused by Klebsiella pneumoniae carbapenemase-producing K. pneumoniae: importance of combination therapy. Clin Infect Dis 55:943–950 22752516 10.1093/cid/cis588

[5] Xu L, Sun X, Ma X (2017) Systematic review and meta-analysis of mortality of patients infected with carbapenem-resistant Klebsiella pneumoniae. Ann Clin Microbiol Antimicrob 16:18 28356109 10.1186/s12941-017-0191-3 PMC5371217

[6] Siu LK, Yeh KM, Lin JC et al (2012) Klebsiella pneumoniae liver abscess: a new invasive syndrome. Lancet Infect Dis 12:881–887 23099082 10.1016/S1473-3099(12)70205-0

[7] Rupp ME, Fey PD (2003) Extended spectrum beta-lactamase (ESBL)-producing Enterobacteriaceae: considerations for diagnosis, prevention and drug treatment. Drugs 63:353–365 12558458 10.2165/00003495-200363040-00002

[8] Paterson DL, Bonomo RA (2005) Extended-spectrum beta-lactamases: a clinical update. Clin Microbiol Rev 18:657–686 16223952 10.1128/CMR.18.4.657-686.2005 PMC1265908

[9] Diekema DJ, Hsueh PR, Mendes RE et al (2019) The microbiology of bloodstream infection: 20-year trends from the SENTRY antimicrobial surveillance program. Antimicrob Agents Chemother ; 63 10.1128/AAC.00355-19 PMC6591610 31010862

[10] Brogden RN, Carmine A, Heel RC et al (1981) Cefoperazone: a review of its in vitro antimicrobial activity, pharmacological properties and therapeutic efficacy. Drugs 22:423–460 6459224 10.2165/00003495-198122060-00002

[11] Matsubara N, Minami S, Muraoka T et al (1979) In vitro antibacterial activity of cefoperazone (T-1551), a new semisynthetic cephalosporin. Antimicrob Agents Chemother 16:731–735 316988 10.1128/AAC.16.6.731 PMC352944

[12] Chiang T-T, Tang H-J, Chiu C-H et al (2016) Antimicrobial activities of cefoperazone-sulbactam in comparison to cefoperazone against clinical organisms from medical centers in Taiwan. J Med Sci 36:229 10.4103/1011-4564.196365

[13] Williams JD (1997) beta-lactamase inhibition and in vitro activity of sulbactam and sulbactam/cefoperazone. Clin Infect Dis 24:494–497 9114205 10.1093/clinids/24.3.494

[14] Akova M (2008) Sulbactam-containing beta-lactamase inhibitor combinations. Clin Microbiol Infect 14(Suppl 1):185–188 18154545 10.1111/j.1469-0691.2007.01847.x

[15] Lin WP, Wang JT, Chang SC et al (2016) The antimicrobial susceptibility of Klebsiella pneumoniae from community settings in Taiwan, a trend analysis. Sci Rep 6:36280 27824151 10.1038/srep36280 PMC5099973

[16] Wang FD, Lin ML, Lee WS et al (2004) In vitro activities of beta-lactam antibiotics alone and in combination with sulbactam against gram-negative bacteria. Int J Antimicrob Agents 23:590–595 15194130 10.1016/j.ijantimicag.2003.10.008

[17] Tseng SH, Lee CM, Lin TY et al (2011) Emergence and spread of multi-drug resistant organisms: think globally and act locally. J Microbiol Immunol Infect 44:157–165 21524608 10.1016/j.jmii.2011.03.001

[18] Xia J, Zhang D, Xu Y et al (2014) A retrospective analysis of carbapenem-resistant Acinetobacter baumannii-mediated nosocomial pneumonia and the in vitro therapeutic benefit of cefoperazone/sulbactam. Int J Infect Dis 23:90–93 24726664 10.1016/j.ijid.2014.01.017

[19] Guclu E, Kaya G, Ogutlu A et al (2020) The effect of cefoperazone sulbactam and piperacillin tazobactam on mortality in gram-negative nosocomial infections. J Chemother 32:118–123 32096456 10.1080/1120009X.2020.1730087

[20] Chandra A, Dhar P, Dharap S et al (2008) Cefoperazone-sulbactam for treatment of intra-abdominal infections: results from a randomized, parallel group study in India. Surg Infect (Larchmt) 9:367–376 18570578 10.1089/sur.2007.013

[21] Lan SH, Chang SP, Lai CC et al (2020) Efficacy and safety of cefoperazone-sulbactam in empiric therapy for febrile neutropenia: a systemic review and meta-analysis. Med (Baltim) 99:e19321 10.1097/MD.0000000000019321 PMC7034635 32080150

[22] Huang CT, Chen CH, Chen WC et al (2022) Clinical effectiveness of cefoperazone-sulbactam vs. piperacillin-tazobactam for the treatment of pneumonia in elderly patients. Int J Antimicrob Agents 59:106491 34871744 10.1016/j.ijantimicag.2021.106491

[23] Weinstein M, Patel J, Bobenchik A, Clinical and laboratory standards institute (2019) Performance standards for antimicrobial susceptibility testing. M 100:148–149

[24] European committee on antimicrobial susceptibility testing (2018) Breakpoint tables for interpretation of MICs and zone diameters. version 9

[25] Chang PC, Chen CC, Lu YC et al (2018) The impact of inoculum size on the activity of cefoperazone-sulbactam against multidrug resistant organisms. J Microbiol Immunol Infect 51:207–213 29037802 10.1016/j.jmii.2017.08.026

[26] Lai CC, Chen CC, Lu YC et al (2018) Appropriate composites of cefoperazone-sulbactam against multidrug-resistant organisms. Infect Drug Resist 11:1441–1445 30237728 10.2147/IDR.S175257 PMC6138961

[27] Chen RZ, Lu PL, Yang TY et al (2024) Efficacy of cefoperazone/sulbactam for ESBL-producing Escherichia coli and Klebsiella pneumoniae bacteraemia and the factors associated with poor outcomes. J Antimicrob Chemother 793:648–655 10.1093/jac/dkae022 38319833

[28] Lee YT, Chiang MC, Kuo SC et al (2016) Carbapenem breakpoints for Acinetobacter baumannii group: supporting clinical outcome data from patients with bacteremia. PLoS ONE 11:e0163271 27644087 10.1371/journal.pone.0163271 PMC5028070

[29] Rho JP, Castle S, Smith K et al (1992) Effect of impaired renal function on the pharmacokinetics of coadministered cefoperazone and sulbactam. J Antimicrob Chemother 29:701–709 1506350 10.1093/jac/29.6.701

[30] Charlson ME, Pompei P, Ales KL et al (1987) A new method of classifying prognostic comorbidity in longitudinal studies: development and validation. J Chronic Dis 40:373–383 3558716 10.1016/0021-9681(87)90171-8

[31] Knaus WA, Draper EA, Wagner DP et al (1985) APACHII: a severity of disease calssification system. Crit Care Med 13(10):818–829 3928249 10.1097/00003246-198510000-00009

[32] Horan TC, Andrus M, Dudeck MA (2008) CDC/NHSN surveillance definition of health care-associated infection and criteria for specific types of infections in the acute care setting. Am J Infect Control 36:309–332 18538699 10.1016/j.ajic.2008.03.002

[33] Nguyen M, Eschenauer GA, Bryan M et al (2010) Carbapenem-resistant Klebsiella pneumoniae bacteremia: factors correlated with clinical and microbiologic outcomes. Diagn Microbiol Infect Dis 67(2):180–184 20356699 10.1016/j.diagmicrobio.2010.02.001

[34] Juan CH, Chou SH, Chen IR et al (2022) Intestinal colonisation with hypervirulent or third-generation cephalosporin-resistant Klebsiella pneumoniae strains upon hospital admission in a general ward in Taiwan. Int J Antimicrob Agents 60:106624 35728713 10.1016/j.ijantimicag.2022.106624

[35] Castanheira M, Simner PJ, Bradford PA (2021) Extended-spectrum beta-lactamases: an update on their characteristics, epidemiology and detection. JAC Antimicrob Resist 3:dlab092 34286272 10.1093/jacamr/dlab092 PMC8284625

[36] Van Boeckel TP, Gandra S, Ashok A et al (2014) Global antibiotic consumption 2000 to 2010: an analysis of national pharmaceutical sales data. Lancet Infect Dis 14:742–750 25022435 10.1016/S1473-3099(14)70780-7

[37] Armand-Lefevre L, Angebault C, Barbier F et al (2013) Emergence of imipenem-resistant gram-negative bacilli in intestinal flora of intensive care patients. Antimicrob Agents Chemother 57:1488–1495 23318796 10.1128/AAC.01823-12 PMC3591916

[38] van Duin D, Doi Y (2017) The global epidemiology of carbapenemase-producing Enterobacteriaceae. Virulence 8(4):460–469 27593176 10.1080/21505594.2016.1222343 PMC5477705

[39] Lee CR, Lee JH, Park KS et al (2016) Global dissemination of carbapenemase-producing Klebsiella pneumoniae: epidemiology, genetic context, treatment options, and detection methods. Front Microbiol 7:184902 10.3389/fmicb.2016.00895 PMC4904035 27379038

[40] Hung MN, Hsueh PR, Chang HT et al (2007) In vitro activities of various piperacillin and sulbactam combinations against bacterial pathogens isolated from intensive care units in Taiwan: SMART 2004 programme data. Int J Antimicrob Agents 29:145–152 16815690 10.1016/j.ijantimicag.2006.02.017

[41] Knapp CC, Sierra-Madero J, Washington JA (1990) Comparative in vitro activity of cefoperazone and various combinations of cefoperazone/sulbactam. Diagn Microbiol Infect Dis 13:45–49 2331849 10.1016/0732-8893(90)90053-X

[42] Gelfand MS, Grogan JT, Haas MJ (1989) In vitro comparison of cefoperazone/sulbactam with selected antimicrobials against 300 bacteroides isolates. Inhibitory activity and time-kill kinetic studies. Diagn Microbiol Infect Dis 12:421–428 2612130 10.1016/0732-8893(89)90113-2

[43] Xiao S, Zhuo C, Zhuo C (2021) In vitro activity of various sulbactam compounds and piperacillin/tazobactam against clinical isolates of different gram-negative bacteria. *Comput Math Methods Med* ; 2021: 1175379 10.1155/2021/1175379 PMC8639252 34868336

[44] Wang Q, Wang Z, Zhang F et al (2020) Long-term continuous antimicrobial resistance surveillance among nosocomial gram-negative bacilli in China from 2010 to 2018 (CMSS). Infect Drug Resist 13:2617–2629 32801799 10.2147/IDR.S253104 PMC7395706

[45] Meatherall BL, Gregson D, Ross T et al (2009) Incidence, risk factors, and outcomes of Klebsiella pneumoniae bacteremia. Am J Med 122:866–873 19699383 10.1016/j.amjmed.2009.03.034

[46] Chetcuti Zammit S, Azzopardi N, Sant J (2014) Mortality risk score for Klebsiella pneumoniae bacteraemia. Eur J Intern Med 25:571–576 24814431 10.1016/j.ejim.2014.04.008

