# Supplementary material for: Multicenter study on clinical outcomes and poor prognostic factors in patients with Klebsiella pneumoniae bacteremia receiving cefoperazone/sulbactam treatment
Source: Eur J Clin Microbiol Infect Dis. 2024 Jul 12;43(9):1777–85. doi: 10.1007/s10096-024-04892-x (PMC11349846; doi:10.1007/s10096-024-04892-x)
Supplement: Supplementary file 1 — Supplementary Material 1 [file 10096_2024_4892_MOESM1_ESM.docx]

Supplemental Table 1.

The minimal inhibitory concentrations and the susceptibilities of cefoperazone alone and in combination with sulbactam with different composites against *Klebsiella pneumoniae* isolates*.*

| *K. pneumoniae* (n=201) | MIC (ug/ml) | | |  | Susceptibility [%(n)]^a^ | | |
| --- | --- | --- | --- | --- | --- | --- | --- |
|  | MIC_50_ | MIC_90_ | MIC range |  | S | I | R |
| CPZ | 0.25 | >64 | 0.0625~>64 |  | 77.61% (156) | 3.98% (8) | 18.41% (37) |
| CPZ/SUL (2:1) | 0.25 | 32 | 0.0625~>64 |  | 89.05% (179) | 1.99% (4) | 8.96% (18) |
| CPZ/SUL (1:1) | 0.25 | 32 | 0.0625~>64 |  | 89.55% (180) | 2.99% (6) | 7.46% (15) |

| CPZnS *K. pneumoniae*  (n=45) | MIC (ug/ml) | | |  | Susceptibility [%(n)]^a^ | | |
| --- | --- | --- | --- | --- | --- | --- | --- |
|  | MIC_50_ | MIC_90_ | MIC range |  | S | I | R |
| CPZ | >64 | >64 | 32~>64 |  | 0.00% (0) | 17.78% (8) | 82.22% (37) |
| CPZ/SUL (2:1) | 16 | >64 | 4~>64 |  | 51.11% (23) | 8.89% (4) | 40.00% (18) |
| CPZ/SUL (1:1) | 16 | >64 | 2~>64 |  | 53.33% (24) | 13.33% (6) | 33.33% (15) |

^a^The susceptibility breakpoints were adapted from Clinical and Laboratory Standards Institute 2019 for cefoperzaone against *Enterobacterales*: S, MIC ≤16 mg/L; I, MIC = 32 mg/L; R, MIC ≥64 mg

CPZnS, cefoperazone-non-susceptible; CPZ, cefoperazone; SUL, sulbactam; CPZ/SUL, cefoperazone/sulbactam; MIC, minimal inhibitory concentration
